# Sarcoplasmic Reticulum Ca^2+^ Buffer Proteins: A Focus on the Yet-To-Be-Explored Role of Sarcalumenin in Skeletal Muscle Health and Disease

**DOI:** 10.3390/cells12050715

**Published:** 2023-02-24

**Authors:** Elena Conte, Giorgia Dinoi, Paola Imbrici, Annamaria De Luca, Antonella Liantonio

**Affiliations:** Department of Pharmacy—Drug Sciences, University of Bari “Aldo Moro”, Bari 70125, Italy

**Keywords:** sarcalumenin, sarcoplasmic reticulum, Ca^2+^ buffer protein, skeletal muscle, sarcalumenin-related skeletal muscle diseases

## Abstract

Sarcalumenin (SAR) is a luminal Ca^2+^ buffer protein with high capacity but low affinity for calcium binding found predominantly in the longitudinal sarcoplasmic reticulum (SR) of fast- and slow-twitch skeletal muscles and the heart. Together with other luminal Ca^2+^ buffer proteins, SAR plays a critical role in modulation of Ca^2+^ uptake and Ca^2+^ release during excitation–contraction coupling in muscle fibers. SAR appears to be important in a wide range of other physiological functions, such as Sarco-Endoplasmic Reticulum Calcium ATPase (SERCA) stabilization, Store-Operated-Calcium-Entry (SOCE) mechanisms, muscle fatigue resistance and muscle development. The function and structural features of SAR are very similar to those of calsequestrin (CSQ), the most abundant and well-characterized Ca^2+^ buffer protein of junctional SR. Despite the structural and functional similarity, very few targeted studies are available in the literature. The present review provides an overview of the role of SAR in skeletal muscle physiology, as well as of its possible involvement and dysfunction in muscle wasting disorders, in order to summarize the current knowledge on SAR and drive attention to this important but still underinvestigated/neglected protein.

## 1. Introduction

Ca^2+^ ions are intracellular messengers essential for signal transduction. In muscle physiology, the main resources of Ca^2+^ for proper functioning of the intracellular Ca^2+^ signals derive from two Ca^2+^ pools: Ca^2+^ sequestered in stores (endoplasmic/sarcoplasmic reticulum (ER/SR)) and extracellular Ca^2+^. The movements of contractile proteins require the correct intracellular level of Ca^2+^ ions to be released from the SR, a specialized membrane system and a component of the cellular reticular network. The presence of these pools guarantees the correct amount of calcium essential for the muscle to perform its pivotal function, i.e., the well-understood excitation–contraction (EC) coupling. EC coupling is a process mediated by mechanical coupling between the dihydropyridine receptor (DHPR) located on the transverse tubule membrane (invaginations of the plasma membrane) and ryanodine receptor type 1 (RyR1) on the SR membrane. Following conduction of impulses through motor neurons (neuromuscular transmission) and the activation of the nicotinic acetylcholine receptor at neuromuscular junctions, muscle membrane depolarization (via propagated action potential) induces a conformational change in the DHPR required for the RyR1 interaction. The latter opens to release Ca^2+^ ions from the Ca^2+^ SR. Subsequently, Ca^2+^ uptake in the SR via Ca^2+^-Mg^2+^ ATPase (SERCA) leads back to a resting level with the replenishment of the SR calcium content [1,2]. Therefore, the maintenance of intracellular Ca^2+^ homeostasis in muscle cells and the correct Ca^2+^ sequestration in the SR are essential requirements for proper muscle contraction. During muscle contraction, resting intracellular calcium (nM range) increases up to the μM range [3]. In addition to the Ca^2+^ released from the SR, this Ca^2+^ increase is obtained through processes involving different molecules and Ca^2+^ transport channels. Among these, it is worth mentioning the entry of extracellular calcium into the cell through store-operated calcium entry (SOCE) via Orai1 and/or the transient receptor potential canonical (TRPC) channels [4,5] or through excitation-coupled Ca^2+^ entry (ECCE). Calcium entry into the cell via SOCE is important for Ca^2+^ replenishment of the SR in order to maintain SR Ca^2+^ content for maximal muscle performance [6], whereas ECCE is a context-specific mechanism contributing to Ca^2+^ entry during muscle contraction [7]. However, free calcium concentration must be rigorously and rapidly buffered for proper calcium signaling and muscle performance. Different Ca^2+^ buffer proteins residing in the cytoplasm (i.e., regucalcin and calmodulin) or in the lumen of the longitudinal and junctional SR of skeletal muscle are believed to regulate EC coupling, SOCE and ECCE processes by binding Ca^2+^ ions (Table 1).

Regucalcin (also called senescence marker protein 30) is a 34 kDa cytosolic multifunctional Ca^2+^-binding protein that lacks the typical Ca^2+^-binding EF motif. It is a marker of aging that principally regulates intracellular Ca^2+^ homeostasis by modulating the activity of several proteins involved in intracellular signaling pathways, such as Ca^2+^ ATPases, calmodulin kinase and PKC [16]. Calmodulin is the best-studied and most highly expressed muscle cytosolic Ca^2+^-binding protein containing two canonical EF-hand motifs that bind up to four Ca^2+^ ions. It mediates Ca^2+^ regulation in a broad range of physiological processes and has numerous downstream targets that are either calmodulin-dependent (i.e., calcineurin, CAMKII, RyR1 and DHPR) or calmodulin-regulated (genes encoding proteins involved in oxidative metabolism, muscle repair and plasticity) [17]. Instead, parvalbumin is a small cytosolic Ca^2+^ buffer protein expressed primarily in fast skeletal muscle fibers. It binds Mg^2+^ when the muscle is in a resting state and dissociates from it and binds Ca^2+^ ions after SR Ca^2+^ release, thereby contributing to muscle relaxation [18].

In regard to the Ca^2+^-buffer proteins residing in the SR, the most abundantly expressed and well-studied Ca^2+^ buffer located in the junctional SR is calsequestrin (CSQ), followed by the calsequestrin-like proteins CLP-150, CLP-170 and CLP-220, as well as HRC, junctate and calreticulin. The most abundant Ca^2+^ buffer protein located in the longitudinal SR is SAR [19]. To the best of our knowledge, most investigations have focused on the main calcium buffer protein, CSQ, while the role of SAR has not yet been fully determined. Nevertheless, the study of the physiological function of SAR and of the potential contribution of its dysfunction to skeletal muscle diseases represents an appealing research field that can reveal hidden biological functions, as well as new therapeutic targets. The present review aims to provide an overview of the currently available research about SAR, focusing on structure, physiological function and involvement in skeletal muscle diseases.

## 2. Overview of Sarcoplasmic Reticulum Structure and the Junctional Ca^2+^ Buffer Proteins: Calsequestrin, HRC, Junctate and Calreticulin

**Figure 1:**
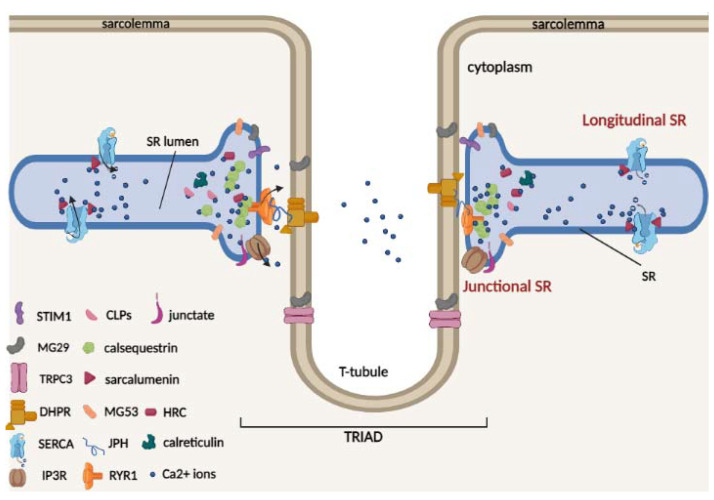
Schematic model of two terminal cisternae on the opposite sides of a central t-tubule (triad) and of the luminal Ca^2+^-binding proteins of skeletal muscle. In the triad, the voltage-activated L-type Ca^2+^ channel dihydropyridine receptor (DHPR) is located on the t-tubule, and the ryanodine receptor Ca^2+^ release type 1 channel (RyR1) is located on the SR. The triad is stabilized by junctophilin (JPH) proteins, which act as structural bridges between the t-tubule and the SR membrane. TRPC3 protein is located on the sarcolemma and can interact with mitsugumin 29 (MG29). The SR/ER Ca^2+^ ATPase (SERCA) pumps are located on the longitudinal SR. RyR1, stromal interaction molecule 1 (Stim1), inositol-trisphosphate receptor (InsP3R), and mitsugumin 29 (MG29) and 53 (MG53) are located in the junctional SR. The Ca^2+^ buffer proteins calsequestrin (CSQ), histidine-rich Ca^2+^ (HRC)-binding protein and the calsequestrin-like proteins (CLPs) are located in the lumen of the junctional SR, while sarcalumenin (SAR) protein is located in the lumen of longitudinal SR.

The sarcoplasmic reticulum (SR) is a specialized membrane system and a component of the cellular reticular network of striated muscle cells that surrounds each myofibril. Two well-defined structural and functional SR regions can be distinguished (Figure 1), namely the longitudinal and junctional SR [20].

The longitudinal SR represents the largest part of the SR in which is located the SR Ca^2+^ ATPases (SERCAs), which pump Ca^2+^ from the cytosol to the lumen of the SR [21,22,23]. It runs along the entire myofibril and connects two terminal cisternae located in proximity of the transverse t-tubules (invaginations of the sarcolemma), forming the “triad”; here, DHPR, which is located on the t-tubules, and RyR1, which is located on the SR, interact physically and functionally. Triad formation is also mediated by junctophilin isoforms (JPH1 and JPH2), which act as structural bridges between the t-tubule and SR membrane, allowing for maintenance of a close and parallel position in the triad junction [24]. JPHs are also able to interact with SR proteins, in particular with RyR1 for JPH1 [25] and DHPR for JPH2 [26], and regulate Ca^2+^ movements in skeletal muscle. Therefore, in the triad, upon membrane depolarization, the DHPR channels undergo a conformational change necessary for RyR1 interaction and activation for Ca^2+^ release into the cytosol and for myofilament contraction. In contrast, junctional SR represents the region of the terminal membrane cisternae that faces the t-tubule/SR membrane [22,24], where RyR1 and other SR proteins involved in Ca^2+^ released from the SR, such as Stim1, inositol-trisphosphate receptor (InsP3R) and mitsugumin 53 (MG53), are located. Furthermore, during muscle differentiation, the formation of triad junctions can be favored by mitsugumin 29 (MG29), a structural protein exclusively expressed in skeletal muscle and localized both on t-tubules and SR terminal cisternae [27,28]. It has also been proposed that in skeletal muscle, the interaction between MG29 and the membrane protein TRPC3 could contribute to regulating Ca^2+^ transients [29].

Luminal Ca^2+^ is bound to high-capacity Ca^2+^ binding proteins within the terminal cisternae and longitudinal tubules [30]. The most abundant skeletal and cardiac muscle Ca^2+^ buffer protein of junctional SR is CSQ, which belongs to the class of high-capacity and medium-affinity Ca^2+^ buffer proteins [31,32,33]. Two CSQ genes are present in striated muscle: Casq1 and Casq2 genes encoding for calsequestrin-1 and 2, respectively. Under physiological conditions, CSQ interacts with triadin and junctin proteins and appears as a monomer [34]. Following Ca^2+^ binding, CSQ monomers polymerize to form large polymers, increasing Ca^2+^ binding ability [35]. In addition to the buffer role of Ca^2+^, both calsequestrin-1 and 2 are important for regulation of Ca^2+^ release during muscle contraction, interacting with ryanodine receptors (RyRs) and contributing to the regulation of Ca^2+^ homeostasis in muscle cells [36,37,38]. Furthermore, CSQ is able to directly interact with muscular STIM1, a key protein involved in the SOCE mechanism, altering STIM1/Orai1 interaction and reducing the refilling of depleted intracellular reticulum Ca^2+^ stores [39,40].

Another minor SR Ca^2+^ buffer protein is HRC, which has structural similarities to CSQ and, like CSQ, binds Ca^2+^ with high capacity and low affinity. It is located within the SR lumen as a multimer [41,42], and unlike CSQ, in the presence of high Ca^2+^ levels, it dissociates from pentamers to trimers and dimers and is less closely folded and more sensitive to trypsin digestion [41]. HRC binds Ca^2+^ directly and could interact with triadin, mediating RyRs activity [42,43,44]. Altered HRC expression is particularly involved in cardiovascular disease [45] and in the onset of gastric and lung cancer [45,46,47]; on the contrary, the increased activity of HRC provides protection against heart damage induced by ischemia/reperfusion [48].

With respect to Ca^2+^ buffer proteins, it is worth mentioning junctate protein, an integral SR membrane protein of 33 kDa. Due to the presence of a negative luminal C-terminal Ca^2+^ binding domain, junctate is able to bind approximately 21 mol Ca^2+^/mol protein [49]. Thus, like CSQ and HRC, junctate contributes to SR calcium storage with a high-Ca^2+^ capacity but low Ca^2+^ affinity. Instead, unlike CSQ and HRC, it has been shown that in HEK cells, junctate is associated with IP3 receptors and TRPC channels, contributing to the SOCE mechanism with its N-terminal domain [50].

Lastly, calreticulin is an ubiquitous 46 kDa Ca^2+^ binding protein located in the lumen of the SR [51]. The structure of calreticulin contains a C-terminal region, which is critical for Ca^2+^ buffering, with an amino acid sequence very similar to that of CSQ [11]. Its conformation is highly dependent on Ca^2+^ concentrations; normally, it is globular, while Ca^2+^ binding causes a change to an α-helical mode [52,53]. Calreticulin mainly functions as a Ca^2+^ buffer protein due to its high binding capacity (20–30 mol Ca^2+^/ mol protein) and as a molecular chaperone, participating in different folding processes of the protein sequence in association with calnexin. The overexpression of calreticulin leads to calcium accumulation in cellular deposits and influences the SOCE mechanism [54,55,56].

## 3. Sarcalumenin Structure and Physiological Functions in Skeletal Muscle

**Figure 2:**
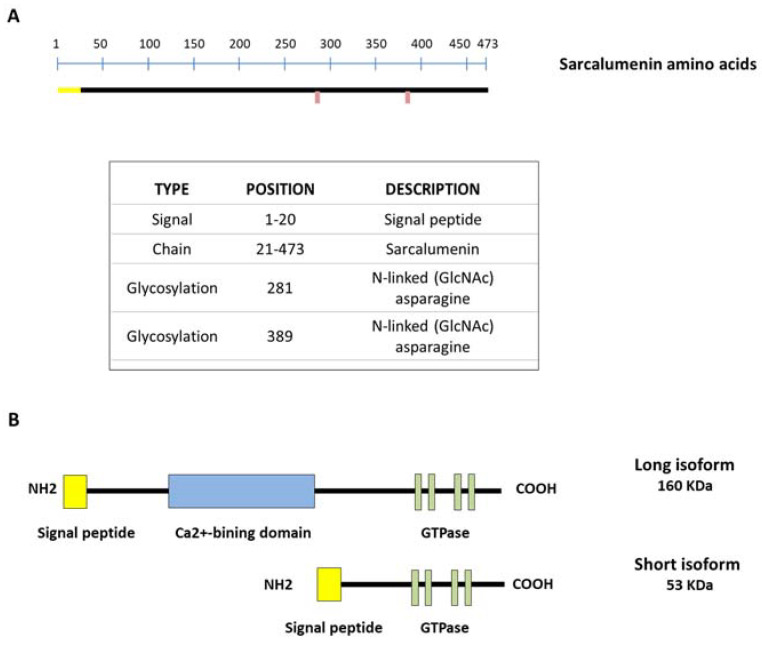
Schematic representation of sarcalumenin (SAR) structure in skeletal muscle. (**A**) Amino acid sequencing of SAR; (**B**) picture showing short and long isoforms of SAR. The long SAR isoform includes a Ca^2+^-binding domain inserted between the N-terminal and C-terminal region, with several nucleotide-binding motifs for P-loop-containing ATPase/GTPase.

SAR is a Ca^2+^-binding glycoprotein composed of 473 acidic amino acids with a molecular weight of 160 KDa (long isoform) first isolated from skeletal muscle by Leberer et al. in 1989 (Figure 2A). However, the SAR gene also encodes a variant of the 53 kDa glycoprotein (short isoform) through alternative splicing of the primary transcript, identical to the COOH-terminal half of SAR and unable to bind Ca^2+^, with a function that currently remains unclear. Both isoforms are predominantly located in the lumen of the longitudinal SR bounding the inner side of the membrane through a Ca^2+^-dependent mechanism. Furthermore, both SAR and the 53 kDa glycoprotein variant represent the major non-junctional SR Ca^2+^-binding protein of striated muscles [9,57]. Only SAR includes a Ca^2+^-binding domain inserted between the N-terminal and C-terminal region, where several nucleotide-binding motifs for P-loop-containing ATPase/GTPase are located [19,57] (Figure 2B). Interestingly, it has been shown that the amount of luminal SAR and 53 kDa glycoprotein variant varies in skeletal muscle depending on the muscle fiber type. In particular, their relative density is lower in slow-twitch versus fast fibers and comparable in gastrocnemius, extensor digitorum longus (EDL) and tibialis anterior (TA) muscles [58]. This different protein expression suggests an adaptation to different physiological Ca^2+^-binding requirements in fast versus slow muscles.

**Figure 3:**
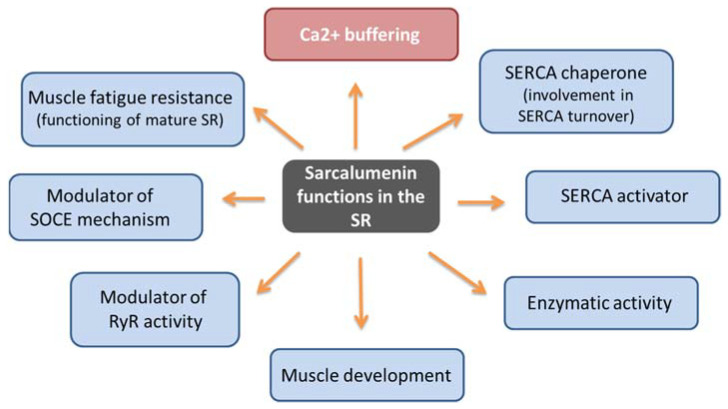
Schematic representation of sarcalumenin functions.

Similarly to CSQ, SAR has a high capacity to bind calcium (35 mol Ca^2+^/mol protein) and moderate affinity (Kd 0.6 mM) [57,59]. It represents the major non-junctional SR protein responsible for Ca^2+^ buffering by acting in the release and uptake of Ca^2+^ and favoring the excitation–contraction–relaxation cycle [19,21,60]. In addition, it has been hypothesized that SAR may have multiple context-dependent functions (Figure 3). First, by performing differential coimmunoprecipitation and chemical crosslinking experiments, it has been demonstrated that SAR colocalizes and interacts directly with SERCA Ca^2+^ ATPase located on the SR [61], suggesting that it performs a maintenance function of the pump itself, also contributing to SERCA turnover by functioning as a SERCA chaperone. Using SAR knockout mice, Yoshida et al. showed that the absence of SAR was paralleled by a reduction in SERCA activity with an unchanged SERCA1 mRNA expression compared with control muscle [19]. The same study also underlined that SAR could exhibit enzymatic activity in SR, considering the presence of the presumed nucleotide-binding motifs for the P-loop-containing ATPase/GTPase in the carboxyl-terminal region. Furthermore, it has been suggested that SAR may play a role in the functioning of mature SR due to increased SAR expression during muscle development [19,62]. It has been also reported that cycles of phosphorylation and dephosphorylation of SAR and HRC by a casein kinase II type could modulate the RyR activity as part of the Ca^2+^-mobilizing machinery during EC coupling [63]. Lastly, SAR could have a role in the SOCE mechanism and muscle fatigue resistance. In particular, using exercised knockout mice for SAR (Sar -/-), Zhao et al. demonstrated that SAR ablation improved both SOCE and the fatigability of exercised skeletal muscles, in correlation with an increased expression level of MG29, a synaptophysin-related membrane protein located in the triad junction of skeletal muscle fibers [59]. SAR could play a role in the SOCE mechanism because the loss of SAR could favor SR depletion, thereby leading to a greater activation of SOCE, an event also favored by the concomitant elevated expression of MG29. Furthermore, it cannot be ruled out that the enhanced resistance to muscle fatigue shown in these mutated mice could be related to compensatory changes in Ca^2+^ regulatory proteins that impact the SOCE mechanism. Thus, in addition to the Ca^2+^ buffer function, SAR can have different and context-dependent functions. In consideration of the limited findings reported on this topic to date, new investigations are needed to confirm and uncover molecular mechanisms underlying SAR function.

## 4. Skeletal Muscle Disorders Involving Sarcalumenin-Mediated Luminal Ca^2+^-Handling Alteration

The elaborate mechanism of Ca^2+^ regulation in muscle cells works with precision. High levels of cytosolic Ca^2+^ are essential for muscle contraction onset, and the reuptake of Ca^2+^ ions is pivotal to trigger muscle relaxation. Defects in the proteins that make up this system and modifications of Ca^2+^ cycling often represent the main cause of neuromuscular pathologies. Importantly, it is necessary to stress that Ca^2+^ SR binding proteins are not simply ion traps that facilitate Ca^2+^ reuptake and increase luminal Ca^2+^ storage capacity but are also multifunctional SR proteins that act as endogenous regulators of Ca^2+^ SR channels and as luminal chaperones [64]. Thus, even small changes in the expression levels of SAR and other Ca^2+^ binding proteins may play an important role in altering the cyclic Ca^2+^ system in skeletal muscle diseases. Here, we discuss current knowledge of the involvement of altered SAR expression and activity in neuromuscular diseases such as Duchenne muscular dystrophy, sarcopenia and malignant hyperthermia.

### 4.1. Duchenne Muscular Dystrophy

Duchenne muscular dystrophy (DMD) is an X-linked lethal disease of childhood that affects approximately 1 in 5000 live births and represents the most frequent neuromuscular disorder in humans [65]. DMD is caused by mutations in the DMD gene encoding the membrane cytoskeletal protein dystrophin [66,67,68]. These mutations cause a loss of dystrophin and lead to progressive muscle degeneration. To date, there are no effective long-term therapies able to provide a lasting abolition of progressive muscle atrophy in humans, although several promising therapeutic strategies have been suggested to counteract the muscle wasting symptoms associated with DMD (i.e., pharmacological, cellular or gene-based therapy approaches) [69,70,71]. The most popular animal model for studying DMD is represented by mdx mice (X-chromosome-linked muscular dystrophy) [72,73], which have a mutation in the dystrophin gene itself, like DMD patients. In this animal model, as well as in DMD patients, the lack of dystrophin results in mechanical instability caused by chronic muscle degeneration and regeneration and a destabilization of sarcolemma [74,75,76] with a high accumulation of macrophages that favor muscle fibrosis [77]. Furthermore, although it is known that the primary abnormality is the loss of dystrophin, several studies have suggested that a rise in intracellular Ca^2+^ due to augmented extracellular Ca^2+^ entry could be an important initiating event in dystrophic muscle and could play a central role in the pathophysiological mechanisms leading to muscle weakness [78,79,80,81,82,83,84]. Increased Ca^2+^ levels may contribute to a cycle of activated protease and Ca^2+^-leak channel activation [85,86]. In addition to the increased cytosolic Ca^2+^ concentration, the Ca^2+^ buffering capacity of the dystrophic SR is also significantly altered [79]. In particular, depending on the analyzed muscle, an increase or a reduction in CSQ levels has been reported [87,88,89]. Interestingly, skeletal muscles of dystrophin-deficient mdx mice showed approximately 70% lower levels of SAR protein relative to wild-type mice [61,87]. A concomitant drastic reduction in SAR expression was also detected in dystrophic cardiac muscle [90]. Therefore, modified Ca^2+^ homeostasis and impaired luminal Ca^2+^ buffering are considered the major downstream effects of sarcolemmal rupture, eventually leading to muscle weakness and accelerating the protein degradation process in dystrophic muscles. All these results corroborate the fact that not only CSQ but also SAR can be considered an important luminal calcium buffer that may play a role in the dystrophic phenotype. Therefore, comprehension of the mechanisms underlying SAR alteration in dystrophic settings could potentially lead to the identification of novel therapeutic targets for DMD.

### 4.2. Sarcopenia

Sarcopenia is an age-related condition characterized by the presence of muscle atrophy and a progressive and generalized decline in muscle strength [91], leading to muscle fragility, loss of muscle mass and augmented fatigue. While loss of muscle mass has a fundamental impact on this condition, progressive muscle weakness is ultimately the primary cause of sarcopenia-associated morbidity and mortality, reducing quality of life in older adults. By understanding the molecular processes that lead to the aging phenotype, it is possible to propose treatments and therapies to reduce the functional impact of muscle weakness and slow down its progression. Multiple factors are involved in the molecular mechanisms underlying age-related sarcopenia, some of which have still not been exhaustively described. Specifically, increased muscle proteolysis, cellular autophagy, altered activation of Ca^2+^-activated proteases/proteasomes and dysfunction of satellite cells have been proposed to be involved [92,93]. Furthermore, several studies propose that impaired Ca^2+^ homeostasis can play a key role in sarcopenia and age-related muscle weakness [94,95,96,97]. Indeed, age-induced uncoupling between DHPR and RYR1 proteins and the consequent decoupling in the excitation–contraction mechanism lead to a reduced Ca^2+^ supply to the contractile apparatus and to a reduction in contractile force [94,98]. Several studies have also shown that a drastic reduction in the Ca^2+^ buffer proteins of the longitudinal SR, in particular SAR protein, occur in sarcopenic skeletal muscle [60,99,100,101]. In particular, O’Connel and colleagues suggest that the significant reduction in SAR expression leads to a reduced capacity of the longitudinal SR shuttle system, which could negatively influence the number of available ions for fast Ca^2+^ release mechanisms, ultimately contributing to a significant decline in contractile force and related muscle function during normal aging [60]. It has been proposed that SAR may play a role in muscle development because a gradual increase in protein expression has been shown during fiber maturation [21]. Therefore, its age-related reduction may have a major impact on muscle progression of the sarcopenic phenotype. Indeed, studies on SAR knockout mice highlight that these mice exhibit phenotypic changes similar to those observed in aged skeletal muscle, including diminished Ca^2+^ uptake into the SR lumen and altered Ca^2+^ handling properties [59], with the only difference being an enhanced SOCE, possibly resulting from chronic adaptation to SAR ablation [19]. Taking these findings into account, it can be suggested that age-induced changes in SAR levels may be due to mechanisms secondary to muscle wasting and that abnormal Ca^2+^ handling related to SAR reduction may contribute to the multifactorial etiology of sarcopenia and could be directly involved in contractile weakness. Unfortunately, very few studies focusing on the evaluation of the role of SAR in sarcopenia are currently available in the literature, and new evidence is needed to confirm preliminary results and to gain more insights.

### 4.3. Malignant Hypertermia

Malignant hyperthermia (MH) is a potentially fatal inherited severe myopathy characterized by a fulminant hypermetabolic state to inhalational anesthetics used during invasive procedures in predisposed individuals [102]. Symptoms of an MH episode are related to an uncontrolled elevation of intracellular Ca^2+^ [103] and include hypoxia, acidosis, hyperthermia, tachycardia, CO_2_ production, hyperkalemia, muscle rigidity and rhabdomyolysis. Mutations in the RyR1 and CACNA1S genes encoding the RyR1 isoform and DHPR, respectively, have been associated with MH [104]. Several dominant RyR1 mutations have been found in MH patients [105], which increase protein opening in the resting state, with a consequent increase in cytosolic Ca^2+^ levels during muscle excitation. These defects favor the onset of the typical signs of MH, i.e., glycogenolysis, ATP depletion, mitochondrial oxidation, lactic acid production, electrolyte imbalance and muscle damage [103]. In addition to mutations in RyR1 and CACNA1S, activated SOCE may contribute to increased intracellular Ca^2+^ levels in the skeletal muscles of MH patients [106]. Furthermore, it was supposed that abnormalities in CSQ and/or SAR might be involved in this disease [58]. Few studies on the involvement of Ca^2+^ buffer proteins in MH pathology are available in the literature, and almost all are focused on the role of CSQ. To date, no differences in the expression of SR luminal Ca^2+^ binding proteins have been found for either CSQ or SAR levels [107]. However, after halothane treatment, premature priming of CSQ for Ca^2+^ release was shown, and considering that RyR1 is CSQ-interdependent, this event favors RyR1 opening in MH muscle [108]. Interestingly, both human patients with mutations in CSQ [109] and CSQ knockouts mice [110] show symptoms similar to those observed in patients with MH, suggesting that the CSQ gene could be considered for genetic screening in MH patients without a mutation in the RyR1 or CACNA1S genes. Although no in-depth study has been published in the literature, the structural and functional similarities with CSQ suggest that SAR may contribute to or favor the onset of MH, suggesting a need for focused investigations in this field.

## 5. Sarcalumenin Ca^2+^ Buffer in Cardiac Muscle

Sarcalumenin is also expressed in the cardiac SR and exerts the same functions described for skeletal muscle, i.e., the regulation of intracellular Ca^2+^ [111]. Importantly, cardiac and skeletal muscle SAR are structurally different. Cardiac SAR shows a distinct electrophoretic mobility, immunological analysis and amino acid sequencing, suggesting that it is a different isoform with respect to skeletal muscle SAR [112]. Furthermore, similarly to skeletal muscle, SAR is able to interact with SERCA2a, improving its stability and modulating its function in the heart [113]. Studies performed using a SAR knockout mouse model have demonstrated that cardiac SAR is pivotal in maintaining the function of the heart by regulating Ca^2+^ transport activity into the SR, even when the heart is subjected to stress. Specifically, biomechanical stress, such as pressure overload, was shown to promote progressive heart failure in this SAR-deficient mouse model, suggesting that cardiac SAR expression is essential for heart adaptation to this stress [113]. Likewise, it was demonstrated that a physiological stress, such as resistance training, was able to reduce the expression of SERCA2a and to alter cardiac function and the maximal exercise capacity of this SAR-deficient mouse model [114]. This evidence suggests that, similarly to skeletal muscle, SAR may play an important role in maintaining cardiac function, particularly under physiological stress, and may be considered an important target to open alternative avenues for potential therapeutic approaches against heart disease.

## 6. Conclusions and Perspectives

Although SAR was discovered 15 years ago as a Ca^2+^ buffer protein located in the longitudinal part of SR muscle, dedicated studies have focused mainly on CSQ, likely due to its higher expression in SR. However, physiologically, SAR represents the major non-junctional SR protein responsible for Ca^2+^ buffering to support and regulate the muscle excitation–contraction cycle. In addition to this function, new roles of SAR have been recently revealed, with multiple context-dependent functions important for the stability of the SR membrane network. Importantly, even small changes in the expression levels of SAR may play an important role in altering the cyclic Ca^2+^ system in skeletal muscle diseases, corroborating the idea of the potential role of SAR as a pharmacological target. It is not clear whether and how the different Ca^2+^ buffer proteins interact with each other or whether protein–protein interactions can occur. Furthermore, the lack of SAR modulators represents a strong limitation. Our current understanding of the involvement of SAR and other Ca^2+^ buffer proteins in skeletal muscle diseases suggests that there may be a relationship between SAR and other SR Ca^2+^ buffer proteins through an adaptive or compensatory response of cells to the alteration of the expression of specific Ca^2+^ buffer proteins by enhancing or inhibiting other Ca^2+^ buffer proteins in a complementary manner. For this reason, it is important to characterize not only CSQ but also SAR, which continues to be underestimated. Once a complete map of the real role and involvement of the two main C^a2+^ buffer proteins, SAR and CSQ (and others), in various pathophysiological conditions has been established, it may be possible to compose a three-dimensional map of each possible protein interaction and fully understand their complexity and involvement in skeletal muscle diseases. Furthermore, studying the interactions between Ca^2+^ buffer proteins and other proteins could pave the way for the identification of ligands and their relative binding sites.

The number of patients with skeletal muscle diseases continues to increase, and improved understanding of their underlying mechanisms is fundamental for the development of new therapies. Investigation of the roles of SAR in skeletal muscle diseases could offer a great opportunity to meet currently unmet needs.

## Figures and Tables

**Table 1:** Summary of Ca^2+^- binding proteins residing in the cytoplasm or in the lumen of sarcoplasmic reticulum of skeletal muscle and their molecular mass and Ca^2+^-dissociation constant. SMP, senescence marker protein; CLP, calsequestrin-like protein; HRC, histidine-rich calcium-binding protein; N.A., not available.

SR Ca^2+^-Binding Protein	Molecular Mass	Ca^2+^ Dissociation Constants	Reference
Calsequestrin	63 kDa	Kd= 1–2 × 10^−3^ M	[8]
CLP-220	220 kDa	N.A.	
CLP-170	170 kDa	N.A.	
CLP-150	150 kDa	N.A.	
Sarcalumenin	160 kDa	Kd = 0.3–0.6 × 10^−3^ M	[9]
HRC	170 kDa	Kd = 1.9 × 10^−3^ M	[10]
Calreticulin	55 kDa	Kd = 2 × 10^−3^ M	[11]
Junctate	33 kDa	Kd = 0.217 × 10^−3^ M	[12]
**Cytosolic Ca^2+^-binding protein**			
Regucalcin (SMP30)	34 kDa	Kd = 0.566 × 10^−3^ M	[13]
Parvalbumin	12 kDa	Kd = 4–9 × 10^−9^ M	[14]
Calmodulin	17 kDa	Kd = 1 × 10^−9^–0.1 × 10^−3^	[15]

## Abbreviations

CAMKII	Calmodulin kinase II
CLPs	Calsequestrin-like proteins
CSQ	Calsequestrin
DHPR	Dihydropyridine receptor
DMD	Duchenne muscular dystrophy
EC coupling	Excitation–contraction coupling
ECCE	Excitation-coupled Ca^2+^ entry
ER/SR	Endoplasmic/sarcoplasmic reticulum
HRC	Histidine-rich Ca2+-binding protein
IP3R	Inositol 1,4,5-triphosphate receptor
JPH	Junctophilin
MG29	Mitsugumin 29
MG53	Mitsugumin 53
MH	Malignant hyperthermia
RYR1	Ryanodine receptor type 1
SAR	Sarcalumenin
SERCA	Sarco-/endoplasmic reticular calcium ATPase
SMP30	Senescence marker protein 30
SOCE	Store-operated Ca^2+^ entry
STIM1	Stromal-interacting molecule-1
TRPCs	Transient receptor potential canonical channels
